# Book Reviews

**Published:** 1833-06

**Authors:** 


### CRITICAL ANALYSES.

Quæ laudanda forent, et quæ culpanda, vicissim,

Illa prius, cretâ; mox hæc, carbone, *notamus.*—Persius.

*A Treatise on the Venereal Disease, and its Varieties.* By Wm. Wallace, m.r.i.a. &c. &c. &c., Surgeon to the Jervis-street Infirmary, Dublin; and to the Infirmary established in that City for the Treatment of Cutaneous Diseases, including Venereal Diseases.—8vo. pp. 382. Burgess and Hill, London.

By those who are best acquainted with the subject, it will be readily conceded that there are still many very important practical questions regarding both the pathology and treatment of that class of disease to which our attention is directed by Mr. Wallace, which admit of additional discussion, and further investigation, than they have yet received from the numerous writers who have preceded him. Something of value cannot fail to be added to our stock of information by every intelligent surgeon who has a fair field for observation of venereal diseases, and who is not so bound to any particular master, or any particular doctrine, as to lead him "to wrest nature from her track," in order to support the theory to which he may happen to be a proselyte. The official situations which Mr. Wallace holds ensure him sufficient experience of venereal diseases, in all their varieties, and the perfect candour with which he treats of every doubtful and disputed point, and the clear account he gives of their symptoms and treatment, demand our approbation, and justify us in recommending his work to the attention of his professional brethren. Impartiality in medical discussions is not often met with; we too generally find writers indulging in *ex-parte* evidence, for the purpose of strengthening their own side of the question; and we fear that, in general, medical works are so superficially glanced over, that the author who has recourse to so unfair a mode of conducting an argument passes undetected and unreproved. In this point of view Mr. Wallace's work will bear, we think, the strictest examination. His object appears to be that which ought alone to influence the mind and guide the pen of every professional writer, namely, a faithful description of what he has himself seen, and a candid examination of the evidence which has been afforded by others.

In the preface, Mr. W. informs us that it was his original intention to illustrate his opinions regarding venereal eruptions by an appropriate selection of delineations, of which he has accumulated a large number. The expense of such an undertaking, and the necessary cost of such a work, would have prevented its circulation among a large class of readers; and he therefore determined to publish the letterpress first, as a distinct work, in the form of a practical synopsis, and afterwards the illustrations, in periodical fasciculi. In conformity with this plan, therefore, the present volume appears, and will be soon followed by another, comprising the remainder of the letterpress, and afterwards by the illustrations, many of which are ready.

This volume includes the whole of the primary symptoms, and these alone; it is complete in itself, and independent of that which is to follow.

In the first chapter the following questions are discussed. Are venereal diseases produced by a specific cause, or morbid poison? or do they arise from common causes of irritation? Are venereal diseases produced by one poison, or by a plurality of poisons? It has been affirmed by one class of pathologists, that venereal diseases are caused by a highly contagious matter, called the venereal poison; that this poison is propagated from the diseased to the sound subject, provided it be placed in contact with a denuded surface, or with those surfaces which are naturally covered by a thin cuticle; that it first produces local disorder, or, in other words, primary symptoms; and, lastly, that this poison sooner or later produces that series of symptoms called secondary, and which appear on parts more or less remote from the seat of the primary disease. On the other hand, it is affirmed that there does not exist any venereal poison, or any specific disease, the consequence of such a poison; that the primary symptoms of venereal diseases are the product of an irritation excited on the living surface by various causes, as mechanical violence, morbid or purulent secretions from the inflamed or ulcerated surfaces of the organs of generation, &c.; that the constitutional symptoms depend on the sympathy naturally existing between all parts of the organization, and particularly between the organs of generation and many other organs and tissues of the body; and, finally, that those diseases which are commonly called venereal are, in reality, several, or various, diseases, arising from various sources, modified by constitution, climate, regimen, treatment, &c. &c. The former opinion was maintained by Astruc and Hunter, and is generally adopted in this country at present. The latter hypothesis is of a comparatively modern date, and has been of late years strenuously supported by several French authors. "It is also the opinion advocated in a work on Syphilis, now in progress of publication in Paris, which, from its magnitude and the number of contributors, may be considered a national undertaking."

Mr. Wallace enters into a very impartial investigation of these questions; and, in our opinion, the arguments he adduces leave us no other alternative than that of fully coinciding with his conclusion, "that venereal diseases are the product of a peculiar morbid poison; for their analogy with other diseases which are acknowledged to arise from morbid poisons sanctions this hypothesis; there are no phenomena opposed to it; and the origin, as well as the characters, of venereal maladies, during their progress, cannot be explained, unless by the supposition that they are the effects of a specific cause." We cannot dismiss this very interesting question without giving the following extracts, which will probably remove a few of the difficulties that some of our readers may feel in agreeing with the author.

"It is affirmed that venereal diseases have, on certain occasions, arisen spontaneously; or, in other words, that they have appeared under circumstances in which the existence of a specific virus could not be suspected. This has been adduced as a powerful argument against the hypothesis of a specific venereal poison. But has it been satisfactorily ascertained that the syphilitic disease does ever appear spontaneously, or under circumstances in which we may not fairly presume the existence of its specific cause? The examination of this question is reserved for a future period; and at present it is admitted, for sake of argument, that venereal diseases may spontaneously arise. But does the apparently spontaneous origin of these diseases overturn the hypothesis of a specific virus? Who can divine the exact nature of the circumstances under which an apparently spontaneous origin of venereal diseases may have occurred? It is by no means impossible for specific morbid poisons, and consequently the diseases which they produce, to arise from the synchronous operation of certain, but hidden causes, although they appear to spring up spontaneously. All contagions, all morbid poisons, must have had an origin. We cannot presume that they have existed from eternity; for, as Mr. Hunter observes, 'animals are not naturally formed with disease, or so as to run spontaneously into morbid actions;' and it is not in opposition to the general laws of nature to infer, that if a morbid poison has commenced at one period from the concatenation or combination of its essential causes, it may also commence in a similar way at other periods. On the contrary, such a supposition is in perfect unison with daily occurrences: for do not certain contagions frequently spring up, and again die away from combined causes, whose mode of action we are not always able to appreciate? It is therefore evident, that from the apparently spontaneous origin of venereal diseases, if such an origin has been observed, no proof in favour of the non-existence of a syphilitic virus can be fairly deduced." (P. 8.)

"It is allowed by all, that we know nothing of any virus or morbific poison, unless by the effects which it produces on the animal economy; for morbid poisons do not, as separate or distinct entities, make any impression on our senses. Therefore, as we can define a virus or morbific poison only by describing its effects, we must inquire what these effects are. Now, while it is admitted that the effects of each specific virus are different, it is also admitted that one common law or general character governs the action of each, viz. the power of producing, when applied under favorable circumstances to the animal economy, a certain effect or disease; which, although variable from the influence of general causes, is always subject, in its progress or development, to laws sufficiently peculiar to distinguish it from all other diseases, and from the effects of all other morbid poisons." (P. 4.)

"*Are venereal diseases produced by one poison, or by a plurality of poisons?*" In direct opposition to those who are inclined to maintain that there is *no* venereal poison at all, may be placed another party, who advocate a *plurality* of venereal poisons with equal zeal and confidence. Some pathologists suppose that the varieties of disease which result from promiscuous intercourse are so dissimilar, and that each variety is, at the same time, so regular in its own phenomena, that they cannot be explained without presuming the existence of a plurality of venereal poisons, each causing its own specific effects. Hunter and Swediaur first propagated this latter doctrine, and it was afterwards extended by Adams and Abernethy. More recently Mr. Carmichael has pursued this subject, and he has formed it into a regular system, according to which there exist four distinct or dissimilar morbid poisons, each producing its own specific effects; and to one or other of these morbid poisons all the varieties of venereal disease are by him attributed, as effects to their causes. All the arguments used in support of the hypothesis of a plurality of venereal poisons are reduced by Mr. W. to the following heads.

"1. Some of the diseases which result from venereal intercourse require mercury for their cure or removal, while others are curable without this remedy, and would often be injured by its employment. Therefore, the former class of diseases must be of a totally different species from the latter, and produced by distinct morbid poisons.

"2. Historical evidence demonstrates that certain forms of venereal diseases existed from the earliest ages, and that to these was added, after the discovery of America, a new and peculiar disease.

"3. Certain diseases, nearly allied in their general characters to each other and to venereal diseases, have been observed in Canada, Scotland, and the West Indies, &c. yet they are considered the product of different morbid poisons; and as these diseases do not differ more widely from one another than the varieties of venereal diseases differ from each other, we have the same reason for supposing that the varieties of venereal disease result from poisons specifically distinct, as we have for supposing that the diseases in question arise from different poisons.

"4. The symptoms of all diseases, which are caused by morbid poisons, are regulated by laws so fixed or determined, that they are always uniform in their appearance and progress; but the diseases which result from venereal intercourse, if considered as the consequence of one morbid poison, exhibit the most dissimilar characters, and must therefore be owing to the action of dissimilar poisons.

"5. The symptoms of venereal diseases, though numerous and varied, form certain determinate groups, in which we observe peculiar forms of primary symptoms followed by corresponding forms of constitutional disease, and the primary and constitutional symptoms of each group resemble one another in their general character, or in their degrees of mildness or severity; while the primary and secondary symptoms of any one group are, in their orgin as well as in their progress, altogether different from the corresponding symptoms of the other groups; and these circumstances cannot be explained, unless by the supposition that each group is produced by its own peculiar specific cause or morbid poison." (P. 14.)

These arguments are separately examined by Mr. Wallace, and, in our judgment, he completely refutes the doctrines of Mr. Carmichael. We condense the replies he offers to them. He observes, in the first place, that experience has shewn that every form of venereal disease with which we are acquainted may be cured without mercury; hence we have no proof that there are two classes of venereal disease, distinguishable from each other by the one requiring mercury for its cure, while the other is curable without this remedy. Those who support the doctrine that a new disease was introduced into Europe after the discovery of America, are compelled to admit that it is impossible to distinguish the symptoms of this new or real syphilitic disease, as they denominate it, from those of the other forms of venereal disease; and they affirm that a line of distinction can be drawn between them only by the influence which mercury exercises over their progress. The third argument may be admitted without injury to the views of those who deny a plurality of venereal poisons. Upon the fourth considerable stress is laid by the advocates of the opposite opinion, and Mr. W. therefore considers it at some length He first remarks, that impressions which result from causes, as far [illegible] we can form an opinion, exactly similar, often produce upon different persons very dissimilar consequences. As an example, the effects arising, in different individuals, from clean punctured wounds are adduced. The characters of vaccina, which are considered specific or pathognomic, are referred to; and it is shewn that they are not so uniform as not to exhibit varieties analogous to those of the venereal disease, and therefore analogous varieties in the appearance of the symptoms which result from the syphilitic virus cannot be considered as any proof of the existence of different venereal poisons.

"It may however be said that the preceding facts bear solely on the primary symptoms, or those produced by the direct application of a morbid poison; and that, although their varieties might be the effect of one morbid poison, we are unable to explain the still more remarkable varieties which are to be observed among the secondary venereal symptoms or eruptions. In reply to this observation, it is only necessary to consider the varieties exhibited by small-pox, which is an eruptive disease produced by a morbid poison, acting through the medium of the constitution." (P. 20.)

"But it may be said, that the effects of syphilis on the skin are sometimes erythematous, superficial, flat, diffused, and scaly, and that these forms differ so essentially from the circumscribed and excavated forms of venereal eruptions, that we cannot suppose the one to be a variety of the other. I admit it to be true that the transition from papular to pustular, or even to tubercular or vesicular cutaneous disease, is more natural and easy than from the erythematous and scaly forms to those of the more circumscribed character, or *vice versâ:* nevertheless, the progress and conversions of many cutaneous diseases satisfactorily demonstrate that such changes may occur among eruptions. Thus pustular impetigo frequently changes to scaly psoriasis. Therefore, as such conversions do occur between these opposite forms of cutaneous inflammations, a similar phenomenon may of course be exhibited by venereal eruptions.

"But, if we return for an illustration to small-pox, we shall find this disease again supporting our views. In fact, the eruption, in certain epidemics of small-pox, has exhibited so much of the diffused or erythematous character, that it has been mistaken for measles. A modern author observes, 'it not unfrequently happens that the papulæ do not rise kindly, but assume the form of stigmatized dots, while the surface is circumfused generally with a brighter or deeper efflorescence, according to the nature of the habit; under which circumstances the disease (variola) makes a near approach to rubeola, and has been mistaken for it.' The same author further adds, that 'in the confluent variety of small-pox, we frequently meet with traits of vesicular and fiery erythema, not unlike in appearance the ignis sacer of the plague;' and Mr. Marshall, in his account of an epidemic small-pox which prevailed in the Kandyan provinces, says there were cases of this disease which assumed a measly appearance." (P. 22.)

As one morbid poison, influenced by extrinsic circumstances, is sufficient to produce all the varieties of small-pox, one poison may, if influenced by similar circumstances, produce analogous varieties of syphilis. Hence it is not necessary to infer, from the varieties observed among venereal eruptions, that there exists a plurality of venereal poisons.

"5. Have the assertions contained in the fifth argument, as detailed at page fifteen, and which constitutes the especial doctrine of Mr. Carmichael, been verified by the experience of other practitioners?

"It is stated, in a circular letter from the army medical department, dated April 2, 1829, and which announces the results of observations made, under the most favourable circumstances for investigation, upon nearly five thousand patients, treated with and without mercury, 'that it appears that no peculiar secondary symptoms are seen to follow from peculiar primary ones; and this general conclusion is unanimously supported by the extensive experience of Messrs. Guthrie, Hennen, S. Cooper, Bacot, &c." (P. 24.)

The worst forms of primary symptoms may occur, without being followed by any secondary symptoms whatever; and mild constitutional symptoms are often produced by the most severe local disease. On the other hand, the most severe secondary symptoms sometimes succeed primary symptoms, which have been so trifling that they have healed without any medical treatment, or so free from pain and inflammation that they have scarcely attracted attention.

"Many facts of a similar kind might be added; but, from what has been said, it is evident that experience denies the existence of that kind of connection which, in Mr. Carmichael's opinion, prevails between the different forms of primary and secondary symptoms of venereal diseases." (P. 25.)

"I admit, that even the occasional sequence of certain forms of constitutional disease after certain forms of primary disease, must naturally lead to the presumption, that there exists some necessary connection, or some common cause of similarity of character. But what is this cause? Is the poison, which has produced each form of primary symptom and its corresponding form of constitutional disease, of a distinct and peculiar species? or is the occasional connection of peculiar secondary symptoms with peculiar primary diseases owing to some cause of modification in the recipient or person infected, which has acted similarly on both the primary and secondary symptoms? After what has been already said, I may fairly leave the reader to decide this question for himself, making first one observation. What are the causes which so modify the effects of the venereal virus, that the same poison, or poison derived from the same person, may produce in different other persons consequences or symptoms of a most dissimilar character? a fact now universally admitted." (P. 26.)

It therefore appears that, although certain forms of secondary symptoms have been occasionally observed to follow certain forms of primary ones, such an occasional coincidence is quite insufficient to establish the hypothesis in question; for, before such an hypothesis could be admitted, it would be necessary to demonstrate that, as far as our knowledge extends, certain primary symptoms are always followed by their own, if by any, secondary symptoms; and that certain secondary symptoms cannot be preceded by any other than by their own primary symptoms. It is also evident that if, on any one occasion, the reverse has been observed, that one occasion would be sufficient to overturn the hypothesis, so far at least as the particular form of disease observed is concerned.

"From all that has been said, we are evidently authorized to conclude, in answer to the fifth argument, that the assertions which it contains are in opposition to general experience; and also that, should a coincidence of certain forms of primary symptoms with certain forms of secondary symptoms be occasionally observed, it would afford no just reason for concluding that such groups are the product of distinct poisons, as the occasional combinations of certain primary with certain secondary symptoms can be easily explained by simpler and more natural causes.

"It is now evident that the question respecting the plurality of specific venereal poisons might be dismissed; but, lest it should be supposed that other arguments of more weight could be advanced in support of this hypothesis, I will proceed further, and demonstrate that it is an hypothesis which is incompatible with several of the phenomena of venereal diseases; and that there are other phenomena, connected with the origin or causation of these maladies, of which it affords no explanation, which would not be the case if it were founded in nature.

"Eruptions of opposite characters, or (more correctly speaking) eruptions which Mr. Carmichael has placed in separate classes, may occur in consequence of the same infection, on the same individual, and at the same time. Thus we are told by Mr. Hennen, in his Principles of Military Surgery, that he has observed the papular, the scaly, and tubercular eruptions united in one person, and the tubercular and the scaly in another. Mr. Lawrence has remarked, in the same individual, the tubercular and scaly forms of eruption. The union of the papular and pustular eruptions is an occurrence of almost daily observation; and we very frequently observe the tubercular and pustular combined.

"Nor is it unusual to witness a succession of dissimilar eruptions in the same patient at different times, and proceeding from the same original infection. Thus, an individual may be attacked by one form of eruption, and in the course of some time that form may disappear, and an eruption of a different character may follow, although he has not received any new infection. Of this description of case we have an example recorded by Mr. Hennen, in which a papular, a vesicular, a pustular, and a scaly eruption, followed the same primary ulcer in the same person, but at distinct intervals.

"It is scarcely necessary to remark that diseases, supposed to be totally different from one another, have been produced in different persons, and even in the same person, by their connexion with the same individual, as such facts are now universally known, and recorded in works which are in the hands of all.

"It thus appears, from what has been just said, that the same infection may produce dissimilar forms of constitutional disease in the same individual, either at the same time or at different times, and also different forms of primary disease in different individuals, or even in the same individual; all of which phenomena are incompatible with the hypothesis that the varieties of venereal diseases, either primary or secondary, are dependant on, or arise from, specific varieties of poisons.

"Again: certain individuals have been observed to contract the same form of disease, and no other, whatever might be the source of infection to which they had been exposed. Thus some persons uniformly get discharges from the urethra, and as uniformly escape ulceration, or *vice versâ.* A case particularly bearing upon this point has been recorded by Mr. Rose, of 'a healthy young man, who was affected with a decidedly sloughing sore on the penis, in consequence of a suspicious connexion. It was not attended with any constitutional disturbance, and yielded readily to mercury. He twice afterwards, at a very considerable interval, had a fresh infection, and the sores each time had precisely the same character with the first.' I have also known many persons, whose primary symptoms were always, and for several times, succeeded by the same form of secondary symptoms. Such occurrences, as well as the analogous ones respecting the primary symptoms, and which, as Mr. Rose observes, are not 'uncommon,' can be explained in only one of two ways. We must suppose that the peculiar disease which uniformly occurred in each individual at different times was owing either to peculiarity of constitution, or to having accidentally received, each time of infection, the same form of morbid poison, out of the plurality which is presumed to exist. I need not point out to the reader the many chances against the second mode of explanation, and consequently that the hypothesis of a plurality of poisons does not explain in a satisfactory manner such occurrences." (P. 28.)

Mr. Wallace enters into a consideration of some other opinions which have been maintained by Mr. Carmichael; but for these discussions we must refer to the work itself.

In the second chapter, a very interesting and careful account is given of "the morbid states, or actions, produced by the direct application of the venereal poison." The symptoms and treatment of primary syphilis form the subjects of the succeeding chapter. We must confine ourselves to the leading practical opinions of Mr. W., which refer to the treatment.

"It may be laid down as a general rule, that the prevention of inflammation and its consequences, of morbid sensibility, and of indolent action, is to be anxiously kept in view during every stage of primary syphilis; for, if any of these morbid states be produced, the case is thereby complicated, and the difficulty of treatment greatly increased.

"It is well known that a certain degree of over-excitement, whether local or constitutional, will cause in one person violent inflammation, its concomitants, and consequences; and that the same degree of excitement in a different person may produce morbid sensibility or irritability; while, under other circumstances, or in other habits, indolent action and induration, &c. may ensue.

"If therefore undue excitement, either local or constitutional, may cause those morbid actions which retard or oppose the cure of primary syphilis, and complicate the indications of treatment, it follows that, during every stage of the disease, great attention should be directed to guard against its occurrence. Indeed, if this all-important principle were carefully acted upon, the disease would, in a vast number of cases, speedily cicatrize without the interference of art. In fact, the closest attention to the great principle of preventing undue or inappropriate excitement should not cease from the commencement of the treatment, until the disease has been brought to a conclusion. Whatever may be the remedies which he is using, the practitioner should be always on the watch, ever fearing the possible occurrence of unfavourable actions; and the moment he remarks those minute changes which point out or foretel the approach of any deviation from the natural characters or progress of the disease, he should pause, and reflect on their cause: in short, he should then modify his treatment, or change it altogether. For it is in accurately observing the very commencement of these changes, and in immediately altering the treatment accordingly, that the skill of the practitioner will be particularly exhibited; and it is the want of this observation, and a blind perseverance in a plan of treatment no longer suited to the individual case, which has led to so many revolutions in practice, and which has frequently caused the most dreadful consequences to result from primary syphilis, which had commenced with the mildest characters.

"Gentleness in handling, unirritating applications, cleanliness, rest, and position, the judicious regulation of regimen, and protection from vicissitudes or intemperance of atmosphere, are the measures for preventing, in constitutions otherwise healthy, this undue excitement.

"It does not appear necessary to speak in detail respecting the employment of these measures, as they are to be used according to the general principles of surgery. It is sufficient to observe, that the practitioner should be more or less particular in the strictness of his rules, according to the nature of each case.

"The directions just given are not, however, to be received as implying that we are never to use such remedies as are commonly denominated stimulants or irritants. These are relative terms; and that application which, at one time or in one case, may act as an irritant or stimulant, may produce, at another time, &c., the very opposite effects. It is therefore merely intended to direct that all applications, of whatever kind they may be, which are found to irritate the disease, or to produce the morbid states above mentioned, or in fact any state which seems to obstruct the progress of cicatrization, are to be omitted, or carefully avoided, the moment that their deleterious influence is observable.

"The treatment suited to primary syphilis varies, according as it may be in the stages of destruction, or in the stages of reparation." (P. 89.)

We seldom have an opportunity of observing primary syphilis before the stage of ulceration; but, "if a case presents itself during the first stage, and while the part is only in a state of circumscribed phlogosis, the diseased structure may without hesitation be snipped off, the wound allowed to heal, and, for security against secondary symptoms, the patient should be treated constitutionally, as if he had not applied until the disease was more advanced.

"It is during the ulcerating stage of primary syphilis, or when the process of granulation has only partially commenced, that our assistance is for the most part sought; and, when the disease is in this stage, there is no doubt on my mind of the propriety or practical utility of immediately applying the nitrate of silver in such a manner as to destroy the diseased surface. I have treated, over and over again, primary syphilitic ulcers with this caustic, and others without it, in the same individual, at the same time, and under circumstances as nearly as possible similar in every respect; and the result has uniformly demonstrated the very great advantage of the former over the latter mode of practice.

"It will be recollected that I am speaking of primary syphilis in a constitution apparently healthy, the disease being in its state of simplicity, and in its stage of ulceration; and if the caustic be applied, under such circumstances, in a proper manner, it will very much shorten the progress of the local disease: it will stop the process of ulceration, and, by preventing in a great measure the necessity of the stage of granulation, it will lead directly to cicatrization. It destroys a surface which seems to have a power of contaminating, for a limited period, continuous parts; and therefore, although actions of reparation would sooner or later spontaneously commence, these actions are hastened by the destruction of the contaminating surface. But, on the other hand, when the process of reparation is already advanced, nothing can be gained, but much injury may be done, by an escharotic application; for, at that period, all the actions are tending to cicatrization, and the destruction of the newly-formed granulations must have the effect of retarding this process.

"I have already alluded to a resemblance in appearance between the ulcer which succeeds the pustule of variola and the ulcer which succeeds the pustule of syphilis. Now it is remarkable, that the application of the nitrate of silver to the pustules of variola, if made within the two first days of the eruption, and in such a manner as to produce a slough, arrests their further progress. This curious fact, which was lately communicated by M. Velpeau to the Académie Royale de Médecine, has been since confirmed several times by myself. I do not however mean, from such results, to derive any direct argument in favour of the application of caustic to the pustules or ulcers of primary syphilis; but the fact ascertained by M. Velpeau is valuable, as demonstrating that the power of an escharotic to control the action of disease caused by a morbid poison is not limited to syphilis; and also that the success or the efficiency of the application of caustic in variola, as well as in syphilis, very much depends on the period at which it may have been used.

"If the disease be in a fit state for the application of the nitrate of silver, this substance should be pointed before using, and then rubbed carefully on every part of the ulcerating surface, (previously cleared, by poultice or emollient lotions, of all incrustations,) until the edge of the ulcer be rendered black, and the surface of a deep-ash colour. But, should any portion of the ulcerated surface have entered on the stage of granulation, that portion is to be avoided, and the application of the caustic confined, as much as possible, to such parts of the sore as are still in the stage of ulceration.

"In making this application, it will be observed, that whatever portion of the sore may be in the stage of ulceration yields much more rapidly to the action of the caustic than such portions as are in the stage of granulation. Indeed, the rapidity with which the vitality of the former is destroyed by the nitrate of silver is very remarkable. This is, however, what might be expected, when we reflect that the ulcerating surface, being *in transitu* to perfect disorganization, must have a power of resistance inferior to that possessed by a granulating surface, which is the seat of reparatory actions. We have also, from this circumstance, a fresh proof of the correctness of the views which have been taken in the preceding pages of the process of ulceration." (P. 92.)

Mr. Wallace does not recommend the caustic to the exclusion of the mercurial treatment; and the objections urged against its application, upon the ground of its not preventing secondary symptoms, are not therefore worthy of attention.

"On the whole, he who opposes the employment of this remedy, under the circumstances above detailed, must have founded his opposition on prejudice or timidity, both of which generally spring from ignorance; or else his powers are unable to distinguish between those cases in which it should be employed and those in which it should not; or having, perhaps, made some injudicious applications of it, he has never afterwards been able to subdue those fears which have resulted from his former ignorance." (P. 99.)

"When the primary syphilitic ulcer has been brought, by the application of the nitrate of silver, &c., to the state above described, or when, from the presence of symptoms already detailed, it appears that the stage of reparation has commenced in every part before we have been consulted, the local and constitutional treatment should be in general essentially mercurial. It is not, however, to be supposed that mercury is indispensable for procuring cicatrization; for, when the primary syphilitic ulcer has arrived at the stage of granulation, the healing process will for the most part advance, though slowly, under the influence of a due degree of appropriate stimulation, or even spontaneously, as I have occasion to demonstrate almost daily at the hospital, when, from the habits of the patient, or from any other cause, it is deemed prudent to refrain from the use of mercury.

"Why then, it may be asked, is mercury employed? I reply, to hasten the process of healing, and to diminish the chance of secondary symptoms. Nor are there many cases, in the whole range of the practice of surgery, which exhibit more remarkably the beneficial influence of medicine. We shall often observe that, in twenty-four hours after mercurial treatment, conducted in the manner recommended hereafter, has been commenced, the appearance of the ulcer will change, and, from being of a dingy yellow, or ash-coloured white, will become of a healthy red; while this alteration in colour will be quickly followed by the process of cicatrization. It is also important to observe, that these rapid changes may be often produced by the local application of mercury alone; but others soon occur which are of equal, or more importance, and are principally, if not entirely, attributable to its action on the constitution. These latter changes are especially denoted by a diminution of the tumidity of the base of the ulcer.

"That any one who has carefully observed the operation of mercury upon a syphilitic ulcer, when this medicine is appropriately employed, should doubt its utility, is to me difficult of explanation. In fact, under the judicious use of mercury, the cicatrization of primary syphilis often follows the stage of ulceration with such rapidity, that there is not time for the reparation of lost parts by the process of granulation, and the ulcer is therefore healed with loss of substance. Whereas, if the sore be left to the unassisted powers of the constitution, the stage of granulation commences as soon as ulceration has ceased, and in general continues until the cavity of the ulcer has been filled to a level with, or raised above, the surrounding parts.

"The power of mercury to suspend, as it were, the granulating stage of syphilitic ulcers, by promoting the stage of cicatrization, is a curious and important fact, not hitherto remarked. Will it not assist to explain, in a satisfactory manner, the cause of some mistakes respecting the phenomena of syphilis into which pathologists have fallen? To allude at present to one: Mr. Hunter considered excavation a character of chancre, and his followers have looked upon this quality as indispensable. But we have found that, although one stage of the primary syphilitic ulcer be characterized by excavation, another is equally marked by the opposite character; hence the one quality may be considered as characteristic of this disease as the other. Yet this last quality does not appear to have been noticed by Mr. Hunter. How then has it happened that Mr. Hunter, so accurate an observer, and so cautious in general in drawing conclusions, was not acquainted with the elevated stage of primary syphilis? I reply, because he was not acquainted with the power of mercury to control this stage, or because he had never allowed the disease to pass through all its stages uninfluenced by mercurial treatment. It would appear that, whenever Mr.

Hunter saw a case of primary syphilis in the stage of ulceration, and consequently of excavation, he concluded that it was syphilitic; and, from the opinion entertained by him of the necessity of giving mercury, he immediately submitted his patient to its influence. This controlled the granulating stage, and produced cicatrization with loss of substance, or without granulation; and as Mr. Hunter evidently had never made himself acquainted with the progress of the primary syphilitic ulcer uninfluenced by mercurial treatment, he not only attributed this mode of healing entirely to the nature of the disease, and not at all to the action of mercury, but, whenever he observed a venereal ulcer in an advanced stage of granulation, he concluded that it was not syphilitic, or that it belonged to that class of disease which resembled syphilis. Nor was his judgment corrected when he observed the primary disease to be followed by secondary symptoms, because he was of opinion that secondary symptoms often followed those primary diseases which resembled syphilis. Indeed, on the contrary, his mind was further riveted in error when these secondary diseases became well without mercury; an event which, in his opinion, could not possibly happen if they were truly syphilitic. How inextricable is the labyrinth into which we are sometimes led by one fatal step; and how indispensable is it to examine, on every occasion, with the most rigid scrutiny, our premises, or the foundation of our reasoning!" (P. 100.)

We regret that our limits prevent us from entering more at length into the various subjects discussed by Mr. Wallace; but we cannot, in justice to him, conclude our notice of his work, without observing, that it contains abundant proofs of sound judgment and extensive experience, and that the surgeon and student will here find a most excellent practical description of the primary symptoms of venereal diseases.

---

*Outlines of Botany; being a Practical Guide to the Study of Plants.* By GILBERT T. BURNETT, Professor of Botany in King's College, London, and Fellow of several Societies. Nos. I. II. and III.—8vo. pp. 174. John Churchill, London.

ENCOURAGED by the example of more than one of our esteemed contemporaries, we sit down, with all due formality, to criticise our own production; and of a truth this practice is not uncommon, although it is not always that an author-reviewer is forced into the confessional.

Some timid persons exclaim "Save us from our enemies!" others, more prudent, "Save us from our friends!" but we find ourselves now compelled to breathe a still more humble aspiration, viz. "Save us from ourselves!"

Few persons have the means of writing a more just criticism upon any work than the author himself, if prejudice did not veil his eyes to the magnitude of errors of which he is conscious, or if the bright spots in the landscape were not dwelt on too constantly, and with too much self-satisfaction. Indeed, an example might be given of one of the most severe critiques ever penned having been written by the author of the work himself.

But, lest we should be blamed for over-severity to ourselves, or (what is more likely) for over-indulgence, we shall not pass any opinion upon the work before us, but let the author speak to our readers as he already has to the public, and let him explain the objects of his undertaking in his own words; and subsequently, if we have room, we will give a few short extracts, as samples of the way in which those parts already done have been performed.

The Prospectus tells us that these Outlines of Botany are intended as a practical guide to the study of plants, and are designed chiefly for the use of students; that the work will consist of several distinct portions, which will be published in separate fasciculi, each containing a condensed account of the principal points in the history, structure, functions, systematic arrangement, geographical and geological distribution, medicinal, dietetic, and other uses of one great natural division of the vegetable world; and, in furtherance of this scheme, the first fasciculus contains "a General Outline of the whole Vegetable Kingdom," introductory to the several classes which are to follow. The history of the first of these classes, the *Flags* or *Algæ*, has likewise now been published; and the history of the *Fungi* will shortly appear.

It is from the Outlines of Algologia that we shall make our promised extracts, only premising that the term is here used in its most extended and comprehensive sense, as including the whole of the Confervæ, Fuci, Lichens, and their allies.

Our extracts will be made quite at random from the different subdivisions of the essay.

"§ 177. Oscillaceæ. The Quick Mosses, or *Quiver-worts*, so called from the vibratory movements or oscillations of their gelatinous fronds, whence indeed is derived their technical synonyme *Oscillaceæ*, form the first type of the section Confervinæ. There are some of them aerial and some aquatic plants; they abound in damp shady situations, in the sea, in ponds, ditches, streams, and even in thermal springs, such as those of Bath, and are of much use in fixing loose sand, and aiding in the deposition of mud. They are as it were the strainers and refiners of nature; for, sometimes rising and floating on the surface, and then sinking through the water to the bottom, they involve, in their filamentous and gelatinous structures, much of the floating refuse matter which they and their allies have been unable to digest as food. Their action may be likened to that of mucilage or isinglass, put by brewers in their vats to refine the beer. And it is by these, and similar plants, that a great deal of mud is not only precipitated from water, but restrained at the bottom of streams, so that rivulets run with crystal clearness over successive strata of offal, which are thus curiously kept undisturbed." (P. 83.)

"*Geographical Distribution of the Confervales.*

"§ 210. From the more equable temperature of the medium in which they live, the range of aquatics is often much less confined than that of terrestrial plants. Water so far diminishes the heat of the torrid, and moderates the cold of the frigid, zones, that several pond and river weeds are known to flourish from the equator to the poles; for example, the European bulrush (Typha latifolia,) has been found in Siberia, North America, Jamaica, China, and the peninsula of Hindostan; our common duck-meat (Lemna minor), which spreads over the whole of Europe, is a native also of North America and Asia, being found in the waters of Pennsylvania and Carolina, as well as in those of Siberia, Tartary, Bucharia, China, Cochin-China, and Japan.

"§ 211. But, although cosmopolites occur amongst those which are usually considered much superior and more perfect plants, it is a curious fact, that there are very few of these inferior grades which seem able to endure equivalent vicissitudes of climate.

"§ 212. Confervæ are comparatively rare between the tropics, and, although not entirely confined to the temperate zones, they become gradually more abundant in the higher latitudes, both of the northern and southern hemispheres.

"§ 213. This is a circumstance deserving especial notice, and the more so, as it is one that could not have been presupposed. Speculation would have suggested that the mud of the Ganges and the Nile, the pools and tanks of Egypt and of India, which swarm with animal life, would not have been less prolific nests of the still simpler forms of plants. But the contrary appears to be the truth; for, whatever allowance may be asked for the less accurate researches that have been made in this department of natural history in extra-European countries than in our own, still the broad fact is sufficiently established: so that, of their comparative paucity in warm countries there can be no doubt.

"§ 214. Connected with the subject of the general geographical distribution of the Confervales, there is a circumstance worthy of remark, not only on its own account, but as indirectly corroborating the statements already made, which, though founded on good evidence, would have been more satisfactory had the examinations been more minute. The hint was first thrown out by Brongniart that no true Confervæ are found in warm springs. This remark has been since confirmed, and I do not know that it admits of any exception; for those Confervales which have been mentioned as inhabiting the Bath and similar thermal waters, belong to the type Oscillaceæ; a group which, it will be remembered, [vide § 181,] verge towards the animal kingdom, and which some naturalists of authority have even wished to exclude from the vegetable reign.

"§ 215. Hence it will appear that the chief geographical range of the Confervinæ, and especially of the types Confervaceæ and Ceramiaceæ, is in the temperate zones; the Confervaceæ abounding both in salt and fresh water; the Ceramiaceæ being exclusively marine.

"As the Oscillaceæ occur in hot springs, along with some of the Ulvaceæ [vide § 241, &c.] that inhabit tropical seas, it is very probable that they will be found, on further examination, to approach nearer to the equator than their allies; but of this no direct evidence has been adduced.

"§ 216. The range of the Nostochinæ appears to be much more extended than that of the Confervinæ; for Palmella, Nostoc, and their allies, are common plants in most temperate regions: while Protococcus abounds not only on the Frozen Mountains, near the North Pole, but is likewise a native of the British Isles, is met with in profusion in the Alpine districts of France, Spain, Switzerland, and Italy, and perhaps even at Paramo, in South America, nearly under the Line [§ 45.]

"§ 217. Whether this plant be indigenous to all these various latitudes, or only a visiter to some, is at present undetermined; but, as it is most permanent and abundant towards the north, and on mountains having a northern altitude, it is likely that its appearance in more southern regions, and in warmer climates, may be owing to occasional migrations.

"§ 218. Of the geographical distribution of the Fragillinæ, far too little as yet is known to allow any generalizations to be ventured; but as many of them are either parasites or epiphytes, and others, as the Globulinaceæ, are for the most part peculiar to certain fluids or solutions, it is probable that their distribution will partake more of a local than a general character; and that they will be found to be more affected by accidental circumstances, always varying, than by the physical constitution of a country, or the vicissitudes of climate." (P. 95.)

"*Geological Distribution of the Confervales.*

"§ 219. The fossil Confervæ of the ancient world appear, by their geological position, as far as it is known, to confirm, in an extraordinary and unexpected manner, the soundness of the conclusions at which naturalists had previously and unpremeditatedly arrived, as to the geographical distribution of the present existing species.

"§ 220. In the first place, they appear to have been much less common in former times than now, for in the older rocks no traces of them have been ever found, and, in the second, to have been unknown during those epochs in which the temperature of the tropics extended further towards the poles. For the vestiges discovered in the upper secondary and tertiary formations are extremely few; and no decided evidence of their existence has hitherto been adduced, even so late as the era of the coal-measures, when ferns, and palms, and pines, were flourishing with the most exuberant wildness; for the filamentary productions found in the schistous deposits of the coal-formations have, as Brongniart well observes, none of the characters of confervoid plants, but resemble more the impressions made by ulvaceous flags, or the aquatic rootlets of still superior vegetables.

"§ 221. The *Chalk-marl*, nearly at the summit of the secondary series, is the first stratum in which vestiges of decided confervoid plants appear; and here only two species, at most, have been found: these have been figured by Brongniart, in his "Histoire des Vegetaux Fossiles," and described under the names of *Confervites fasciculata*, and *C? Ægagropiloïdes*. The first bears a very considerable resemblance to our present species, *C. ærea* [§ 175, fig. D.], and the latter to our moor-ball, *C. ægagropila* [§ 184, fig. A.], or rather to our sea-balls, *Ægagropilæ marinæ*, which are formed by the aggregation of the leaf-fibres of Caulinia oceanica. Hence a query is affixed to its generic name; for, no articulations being perceptible, it is a very doubtful Confervites.

"§ 222. In the tertiary series another species has been discovered, [§ 221, fig. E. F.], which Brongniart calls *Confervites Thoreæformis*, from its similitude to certain species of the recent genus *Thorea; e.g.* to the T. *ramosissima* of France, or rather to the T. *violacea* brought by Bory St. Vincent from the Isle de Bourbon. Brongniart states this to be the most satisfactory example he has seen of a fossil Conferva: the specimen from which his figure was taken is preserved in the collection of the Marquis de Dré.

"§ 223. The above-named two species of Confervites are all that have been hitherto discovered and absolutely determined; hence there are not sufficient materials collected to decide how far the various sections of the Confervales could be distinguished, if found in a fossil state. Such a distinction would certainly be difficult; Brongniart thinks almost impossible; at any rate, it would be useless now to subdivide so small a group: therefore, it is agreed that all articulated filamentous fossils shall for the present be associated together, and form the genus *Confervites*, which, should a greater number hereafter be discovered, may become the common name of the fossil section, equivalent to *Confervinæ* among recent plants.

"§ 224. That further researches will enlarge the group there can be little doubt, for Brongniart mentions having examined, in a collection at Verona, various fragments of marine fossils bearing the impressions of articulated plants, apparently Confervites, of several different species. One fragment, he says, seemed, from the rounded granules towards the ends of the filaments, to bear the impression of a plant similar to some of our modern *Ceramiaceæ*, and several others which were in too imperfect a state to be specifically described, he considered to be associates of the various genera of the same type." (P. 97.)

"§ 225. Thus the species of this order, which, by their fossil remains, have been decidedly recognised as denizens of the ancient world, are but two, and, even including the doubtful and undetermined markings, the amount still remains very small. So that, either all vestiges of these plants, if they were formerly as abundant as they are now, must have been wiped out, which there is no reason for supposing, or the physical condition of this planet must formerly not have needed the services they now perform; or have been at one time incompatible with their existence, and at another unfavourable to their increase.

"§ 228. Thus the geographical and geological distribution of the Confervales curiously coincide, and the facts collected on either hand as curiously confirm each other. For, as in our own time, these plants abound in temperate regions, and are unknown, or few, in warmer latitudes, so likewise in former eras, when, from other evidence, it is believed that the temperature of this globe was higher than at present, geological researches affirm that they were, in like manner, either absent, or as scantily produced.

"§ 229. Thus is the first link of an astounding chain of testimony secured; for, from the beginning, there were natural witnesses of Nature's works, and natural records kept; and these humble plants will perhaps afford one of those scattered sybil-leaves, which, if rightly arranged, may unfold, in part, the ancient history of the world." (P. 99.)

The *Fucales* we pass wholly without extract, as we cannot afford more room than for a short notice of some of the *Lichenales* found on the officinal barks.

"§ 384. Until the publication of Fee's Memoir on the Cryptogamic Epiphytes of the Officinal Barks, the study of the opegraphas and their allies seemed to be one rather of speculative amusement than of practical utility. But now the case is wholly changed, since these graphic plants, these living letters written by Nature's hand, are shewn to constitute inscriptions legible by men. Always curious indeed, and admirable, even to the least tutored eye, did the examination of these mimic characters appear; and as fancy traced the likeness to various oriental signs, so were these little plants called scripture-worts, some Hebrew (Opegrapha hebraica), some Chinese (Arthonia sinensigrapha), and so forth. But, like the hieroglyphics of the Egyptian fanes, their meaning was buried in obscurity, and so little guessed at, that it often was doubted whether they had any secrets to reveal. They were sources of wonder rather than of wisdom, until the Young and the Champollion of the vegetable world arose, and by means of a natural Rosetta-stone deciphered these hitherto unknown manuscripts, and taught us to peruse this part of the sacred Scriptures of creation." (P. 158.)

"§ 391. *Chiodecton*, the snow-wart, so called from χιὼν and δεκτικος, the tubercles being seated on a thallus, white like drifted snow, promises to become an important diagnostic sign of the bark of the Cinchona cordifolia, to which one species (C. effusum?) is said by Fee to be peculiar.

"§ 392. *Pertusaria*, the porelet, (from *pertusus*, perforated,) and its ally, *Thelotrema*, (from θηλη, a nipple, and τρῆμα, a hole,) are further illustrations of this subtype. They are chiefly interesting as affording two further distinctive signs of officinal barks, the latter, (T. urceolare, of the Cinchona oblongifolia, or red bark), the former in that variety of *P. communis* called amara, (*Variolaria amara* of some writers,) of the inferior value of the barks on which it is found. According to Fee, this lichen chiefly grows on the very old bark of Cinchona cordifolia, and denotes by its presence a bad quality. *Variolaria amara* is itself, as its name imports, extremely bitter; and, as it is very abundant in many districts, it ought, as the same lichenologist observes, to have its medicinal properties ascertained, for, probably, it might be employed with advantage in certain diseases; it readily imparts its intense bitterness both to water and spirit." (P. 160.)

"§ 396. *Verrucaria* and its allies, forming the small subtype *Verrucaridæ*, chiefly differ from the Endocarpidæ, to which, with the exception of their proper excipuli, they are very similar, by the non-dehiscence of the apothecia, and the nucleus being deliquescent. The species, both European and exotic, are very numerous, and a great many are epiphytic on the officinal barks; but in this respect they are such cosmopolites, that they do not afford any very decided differential signs, or at least the distinctions have not as yet been sufficiently made out. One species of *Ascidium*, which grows commonly on several of the officinal barks, may be given as an illustration.

"But, although these and many other epiphytic lichens do not each indicate specific plants, their general presence or absence will often assist in discriminating otherwise nearly similar vegetable productions. For example, it has been already mentioned [§ 365,] that the beech, though not absolutely destitute of lichens, is far less licheniferous than the oak, the ash, the hawthorn, or the fir; and in like manner it has been found that the spurious angustura bark, which is obtained from a species of *Brucea* (B. antidysenterica,) and which contains a poisonous principle analogous in its properties to *Strychnia*, bears very few lichens of any kind; while the true *Cusparia febrifuga*, which is an admirable tonic, bears them in abundance." (P. 162.)

"§ 401. *Rhizomorphaceæ*. The *Rootmoss*, (Rhizomorpha,) so called from the radiciform elongations of its thallus, gives name to the *Rhizomorphaceæ*, the first type in the section *Byssinæ*.

"§ 402. The several species of the genus *Rhizomorpha*, such as *subcorticalis*, *divergens*, *subterranea*, *phosphorea*, &c., are very common on the trunks of dead trees, especially beneath the bark of firs; in cellars, particularly wine-cellars, among the saw-dust; in lead and coal mines, and other similar situations; thus shewing, by their vegetating in the dark, their secession from the normal lichens.

"§ 403. These plants are active agents in decorticating dead trees, and assisting in the disintegration of lifeless organic bodies, which would otherwise encumber the surface of the earth, and be exceedingly tedious in their removal. Portions of bark several feet in length, or even extending from the base to the bole of a large tree, will, when the Rhizomorphæ locate themselves between them and the wood, soon become so far loosened as either to fall off spontaneously, or to be unable to resist the slightest external force; and trees thus debarked are quickly preyed upon by fungi, and other wood-destroying plants and animals.

"§ 404. Several species, especially *subcorticalis*, *subterranea*, and *phosphorea*, are occasionally phosphorescent, and more or less luminous in the dark; and hence they often give to the cellars and mines in which they grow an extraordinary and brilliant appearance. In the coal-mines in the vicinity of Dresden they are said to be so abundant and so luminous, as even to dazzle the eye by the brilliant light that they afford. This light is increased by the warmth of the mines; so that, hanging in festoons and pendents from the roofs of the various excavations, twisting round the pillars, and covering the walls, they are said, by their brightness, to give to the Dresden coal-mines just mentioned, in which they abound, the semblance of an enchanted palace. Mr. Erdman, the commissioner of mines, thus describes the appearance of the Rhizomorphæ in one he visited: "I saw the luminous plants here in wonderful beauty; the impression produced by the spectacle I shall never forget. It appeared, on descending into the mine, as if we were entering into an enchanted castle. The abundance of these plants was so great, that the roof, and the walls, and the pillars were entirely covered with them, and the beautiful light they cast around almost dazzled the eye. The light they give out is like faint moonshine, so that two persons near each other could readily distinguish their bodies. The lights appear to be most considerable when the temperature of the mines is comparatively high."

"§ 405. The type Rhizomorphaceæ, characterised by having the sporidia internal, includes, according to Fries, two subtypes, in the first of which the sporidia are contained within a pseudo-perithecium, formed by the collocation of the turgid fibres of the thallus. The thallus likewise, in this subtype, is continuous, radiciform, of a dark colour verging to black, and formed of many filaments blended into a common stratum. Rhizomorpha is the normal genus, and hence it is called the Rhizomorphidæ. Ascophora seems a doubtful ally, although by Fries referred to this group.

"§ 406. The Rhizomorpha cinchonarum is a rare species, but, whenever found, it is a sufficient indication of the worthless state of the barks it grows upon, demonstrating by its presence that their medicinal qualities are much impaired, if not entirely cancelled by putrescency." (P. 163.)

*Outlines of the Course of Lectures on Military Surgery, delivered in the University of Edinburgh.* By Sir George Ballingall, M.D., F.R.S,E., Regius Professor of Military Surgery, Fellow of the Royal College of Surgeons; Surgeon Extraordinary to the King; one of the Surgeons to the Royal Infirmary, &c.—8vo. pp. 589. Black, Edinburgh; Longman and Co., London.

The course of lectures on Military Surgery, of which we have in this volume the "Outlines," deserve the especial attention of surgical students who are destined for the army, and cannot be perused without advantage by any practitioner, in whatever station he pursues his professional avocations. To the former class, of course, these lectures will be peculiarly acceptable; for, although it is true that the studies of the military and civil surgeon must be, to a great extent, pursued with the same view, yet there are many topics which the one should be acquainted with that the other may fairly pass over, and which are not discussed in the general works on the practice or science of surgery. All such subjects which appertain almost peculiarly to the duties of the military surgeon are considered by Dr. Ballingall: many of them, indeed, are briefly dismissed, but still upon each much interesting information is given.

In the first chapter there are many valuable hints upon the important duty of selecting and examining recruits for the army. We have often had to perform this task, and remember how very acceptable would have been to us the information given by the author. It is now, we believe, universally admitted that the strength of an army is best supplied by the recruits from the country, rather than from towns or large cities.

"This view of the matter is strongly confirmed by a statement contained in a very interesting paper on the Health of the Peninsular Army, by Sir James M'Grigor, published in the sixth volume of the Medico-Chirurgical Transactions of London: 'Of the classes of society from which soldiers are recruited, I believe it will be found that, *cæteris paribus*, tradesmen and manufacturers, particularly those from large towns, are soonest swept away by the fatigues and diseases of an army; and that those who have followed agricultural pursuits are the most healthy. Three hundred and fifty-three recruits joined the 7th regiment in Portugal, in the years 1810-11. Of these, two hundred and one were artificers and manufacturers, and one hundred and fifty-two had followed agricultural pursuits. In the course of a few months, one hundred and twenty-two of the former died, and sixty-two of the latter; the proportion being six out of ten in the former case, and four out of ten in the latter.'" (P. 31.)

In the opinions just quoted Dr. B. fully concurs.

"On the subject of stature and of bodily conformation, it may be observed that crowned heads seem, in general, to have a predilection for men of lofty stature and imposing appearance; and what are termed the household-troops in this and other countries consist of men much beyond the average height; but such men frequently owe their superiority to an additional length of limbs, and are often found to have defective chests, very disproportioned to the bulk of their extremities. This renders them, particularly in our variable climate, subject to pulmonary diseases; and it has been stated to me in conversation with the surgeon of the Blues, one of the most splendid regiments in Europe, that his men are particularly liable to suffer from such complaints. Tall men are said to be more subject to diseases, particularly those of the chronic class, than men of a medium size, and they are frequently the first to fail under fatigue; men of this description therefore are not the most eligible for the general run of military duties, and 'in the present time, when the fate of battle is often decided by firearms, to which the hand of a man of six feet gives no more impulse than the hand of a man of five, it is not easy to see the reason of the rule which so generally influences the choice of those who select subjects for the formation of armies.'

"The age at which soldiers are enlisted is a point of much importance, and does not appear to have always met with that attention which it merits. Upon the principle of inuring men from an early age to those pursuits in which they are subsequently to be employed, it is generally thought that we can scarcely enlist men too young; there is nothing, however, so mysterious in the duties of a soldier as to prevent a man, possessed of the necessary physical powers, from learning them at almost any period of life; while, on the other hand, by enlisting boys before their growth is completed, and their constitutions formed, it is quite impossible to foresee whether they will ever attain those physical powers necessary to capacitate them for the duties of a soldier; some of them will perhaps turn out better than we expect, but many of them will also, in all probability, turn out worse, and will ultimately prove a loss to the service, or what are termed in the army 'the king's hard bargains.'" (P. 32.)

The most eligible period of life for enlistment Dr. B. considers to be from twenty to twenty-five years of age. He touches but briefly on the physical defects which ought to lead to the rejection of recruits. They are specified in the printed instructions furnished to every surgeon, and their enumeration in this work is therefore the less necessary.

The diet, clothing, and exercise of troops, are subjects which demand the strict attention of the surgeon; for, although he cannot exercise an absolute control over either, it will be expected that he should furnish hints and information respecting them for the consideration of the superior officers, who rarely fail to pay a proper degree of attention to the recommendation of the medical officers upon points on which their judgment may be so advantageous to the soldiers, and of so much importance to the country. The food of a soldier may be coarse, but it should always be wholesome, nutritious, "and I should say abundant, although a distinguished military writer has dwelt upon the advantages of inuring soldiers to habits of abstinence." Dr. Jackson observes, that

"Dignity of mind and real military virtue have no connexion with sumptuous living. The conqueror is ordinarily frugal and homely; the conquered is frequently rich, luxurious, and what is called refined. It is reported of General Wolfe, who, while a man of superior goodness, was perhaps the most perfect soldier of the age in which he lived, that the cook and butler did not much engage his attention. He never gave an elegant, and rarely an eatable dinner to persons of the *haut gout;* the epicurean was disgusted, the soldier was regaled. General Wolfe's table was said to be an epitome of a Spartan messroom; no one rose from it without having been furnished with the opportunity of carrying away a military lesson, and few left it without feeling an accession of military importance communicated to the mind by the impressive influence of a hero's spirit."

One of the principal causes of the moral crime and insubordination both in the army and navy, is doubtless owing to the pernicious habit of dram-drinking. To check this mischievous habit every effort should be made, both by the officers and surgeons; and experience has shewn that, when the men become fond of tea, they are weaned from drinking strong liquors to excess.

The clothing of the soldier is a subject of much importance to their health and comfort, and does not escape the attention of the author.

"In some instances, the very mode of cleaning the soldier's clothing has been a means of inducing disease; thus the cleaning of the white trowsers with wet pipeclay, and putting them on before they are thoroughly dry, is apt to occasion rheumatic attacks, of which I have seen numerous instances. It therefore becomes the surgeon's duty, by his personal remonstrances and representations to the officers, to do everything in his power to check a practice so likely to hurt the health of his men. The use of pipeclay indeed ought strictly to be confined to the belts, gloves, and other leathern appointments, and ought not to be employed so much as it is in cleaning other parts of the dress; it is at the best (as a facetious friend of mine used very emphatically to express himself,) only putting a quantity of white dirt over a quantity of black dirt." (P. 45.)

The medical officer is not unfrequently consulted respecting the comparative advantages of different situations for the accommodation of troops in camp, in barracks, or in billets, and he should of course possess sufficient information upon the subject.

"The situation of a camp must sometimes be regulated by accidental circumstances, over which the opinion of a medical officer can have little control; but whenever a choice of the ground for encampment is submitted to professional opinion, it will be proper to recommend a dry elevated situation, remote from marshes, swamps, stagnant waters, and from the immediate neighbourhood of orchards, forests, or underwood, which are calculated to retain moisture. A camp is most advantageously situated on a gentle declivity, on a dry soil, and in the vicinity of a running stream. In order to ascertain the state of the ground, it may sometimes be necessary to dig into it to some extent; for, although apparently dry on the surface, it may be found sufficiently wet to the depth of a few feet; and if so, ought, if possible, to be changed, particularly if an encampment is to be stationary. A camp should never be formed on ground recently occupied, nor on a field of battle where much carnage has recently occurred. Many favourable spots for encampment are to be found on the banks of rivers, which perhaps, upon the whole, afford the most eligible sites. We must yet bear in mind that, when the banks of the river are low, or the country subject to periodical rains, or sudden inundations from the melting of snow on contiguous mountains, there may be a very serious danger from this cause. Against the danger of such a position we are cautioned in Mezerey's 'Médicine d'Armée,' where he states a case in which the Austrian army lost five hundred men and two hundred horses, from a sudden inundation of this kind.

"When an encampment is inevitably situated in a low or unfavourable position, the kindling of large wood-fires in the windward parts of the camp is recommended by Mindererus, in his 'Medicina Militaris,' as contributing to preserve the health of the troops; and, with the same view, discharges of artillery, and bonfires in the streets, have sometimes been adopted in sickly garrisons, for the pnrpose of purifying the atmosphere; but the heat which they occasion, and the terror which they inspire, render their efficacy very questionable. Various modes of ventilating tents have been recommended, some of which are described in a little work entitled 'The Soldier's Friend,' by Mr. Blair, formerly surgeon of the Lock Hospital, in London; but, as none of these plans have ever come into general use, nor indeed have been found requisite, it is unnecessary to enlarge upon them here. The most obvious and perfect way of thoroughly airing the tents is by striking them occasionally, and exposing the straw, blankets, and soldiers' clothing to the open air; the necessity of frequently changing the straw, and enforcing cleanliness in camp in every possible way, are circumstances too obvious to require any effort of reasoning to enforce. With this view, the slaughtering of cattle, and every thing likely to create noxious or putrid effluvia, ought to be conducted without the camp, and on the side of it opposite to that from which the wind generally blows." (P. 49.)

In the construction of barracks, the two great objects to be kept in view are the means of *thorough ventilation* and *perfect cleanliness.*

"The essential part of the mode of ventilation which I would recommend, is to convert the passages, lobbies, and staircases of a barrack, or public building, into spacious air-trunks, or reservoirs, communicating directly with the external atmosphere, and supplying the rooms adjoining them on either side, both by means of the doors opening into such passages, and by means of additional apertures made for the special purpose of ventilation. Thus, at either extremity of the passages, there should be a window reaching from the ceiling to the floor of each story, the upper part capable of letting down, and the lower of lifting up, so contrived, however, as not to shut perfectly close at either top or bottom, but to leave a slit or aperture at least two inches wide, forming at all times a direct communication with the external atmosphere; and in fine weather these passage-windows may remain open to any extent, giving the most unlimited access to the external air. In the floor of each passage one or more apertures should be made of at least two feet square, covered with strong iron gratings, and in the roof a properly sheltered aperture or penthouse, so as to admit the escape of the air outwards, while it effectually prevents the entrance of snow or rain. By this direct and perfect communication of all the passages with the external atmosphere, as well as with each other, a constant supply of pure and unrespired air will exist within the building. To place the passages thus prepared in action as ventilators, there should be placed over each of the doors opening from them into the adjoining apartments, a Venetian window reaching to the ceiling, while the door itself should not reach within an inch of the floor; but the principal dependence for a uniform supply of air should rest on means independent of the ordinary openings, and may be effected in the following manner. Into each room, on a level with the floor, let apertures be made of six or eight inches diameter, and from ten to fifteen feet distant from each other, communicating with the passage or main air-trunk; and the same description and number of apertures are to be made close to the ceiling corresponding with the unbored spaces of the lower range of air-holes, it being observed that this upper row of perforations must be conducted through the exterior wall of the building, communicating directly with the external air, and so disposed as to prevent the access of rain or snow. By this means we provide for a constant supply of unrespired air from the passages or reservoirs by means of the lower range of perforations, while the respired and heated air is permitted to escape by the upper range." (P. 54.)

Dr. Ballingall states that, during a campaign, we ought not to calculate upon less than ten sick for every hundred fighting men; and this number is often fearfully increased if the army is very numerous, and much concentrated; if it is encamped on wet ground; if it has experienced great privations; and, finally, if it is discouraged by defeat, or want of confidence in its chiefs.

"An army of 100,000 men may expect, according to Vaidy's calculation, to have 10,000 sick during a campaign, independent of any rencontre with the enemy; of which number 5000 or 6000 may be medical, and the rest surgical cases; but, after a battle, the proportion will be reversed; and, under the most favorable circumstances, he calculates upon 10,000 or 12,000 wounded, in addition to the above. To this we have sometimes to add the number of wounded left in our hands by a vanquished enemy, to whom our cares are equally due. These calculations become of great importance to medical officers, particularly as they rise upwards in the gradation of ranks, and come eventually to be consulted as to the extent of the necessary establishments for the sick, or to regulate the proportion of medical staff and hospital stores to be allotted for any given service." (P. 66.)

Dr. B. makes many useful comments on the subject of military hospitals; and mentions the leading points to which the surgeon should direct his attention, when he is consulted as to their plan of construction. The best means of transporting the sick and wounded from one place to another, are also described.

The preliminary sections of the work, to which we have briefly alluded, all contain much interesting information. In those which follow, the surgical diseases of soldiers and seamen are discussed, and their nature and treatment accurately and clearly described.

"*Punishments.* There are perhaps few situations of greater professional responsibility than that in which a military surgeon is placed, when called upon to regulate the extent to which corporal punishment may be inflicted on a soldier without endangering his health or the interest of the service; without, on the one hand, weakening the ties of military discipline by undue lenity, or, on the other, sanctioning an extent of punishment which may permanently injure the individual, and thus prove a heavier infliction than the law contemplates.

"Upon such punishments as merely imply the degradation or disgrace of a soldier, it is not within my province to enlarge, and the following observations must be confined to those in which his health is involved. Many modes of punishment formerly in use in the British army are now happily abolished, and those still permitted are specifically described; so that there is little chance of those cruelties being revived which have sometimes been practised, even in recent times, by arbitrary and capricious individuals, when serving upon distant foreign stations, removed from the surveillance of their superiors, or the salutary control of public opinion." (P. 518.)

Flogging is now greatly restricted. A regimental court-martial can inflict only two hundred lashes; a circumstance which relieves the surgeon from much irksome duty, and heavy responsibility; but, as the regulations of the service still provide for the corporal punishment of offenders, they also enforce the attendance of the surgeon, to see that it shall not be carried to excess.

"In this climate, soldiers are generally able to bear the limited number of lashes to which the sentence of regimental courts-martial has latterly been restricted; but on foreign stations, men's constitutions are sometimes brought into a state in which even a hundred lashes may prove most seriously hurtful. The formidable ulcers formerly noticed as prevalent upon some of the East-India stations are liable to supervene upon punishments; and I have now before me some remarkable cases of this kind which occurred amongst soldiers who had served on the western coast of Africa. In all climates, it should be recollected, that those individuals who are most likely to render themselves amenable to corporal punishment, are very frequently the least able to bear it; and this applies equally to those whose vital powers may be temporarily impaired by an occasional debauch, and to those whose constitutions may be more seriously and permanently injured by a long-continued course of dissipation in early life, and by the remorse and broken spirit under which they are sometimes brought to punishment. This applies particularly to those wretched individuals, the profligate irreclaimable sons of gentlemen, who, after having spent their patrimony, offended their friends, and estranged themselves from society, enlist in the army. Of the unfavorable state of such subjects for military punishments, I shall never forget a remarkable instance which occurred to myself at the very commencement of my service as an army surgeon. The patient was a near relative of a wealthy Jew merchant; he had dissipated his patrimony, subsequently enlisted, and then deserted; he was overtaken before he had got many miles from the garrison, was brought back a prisoner, instantly tried by a garrison court-martial, and was punished with more than usual solemnity, by the tap of the drum. Although this individual did not receive what at that time was considered a severe punishment, yet 'he never looked up after it,' but was taken into hospital in a state of great mental and bodily depression, his appetite failed, and he became much emaciated; although, fortunately for the credit of the surgeon, his back cicatrized, yet he was seized with dysenteric symptoms, and in a few weeks died. To add to the melancholy catastrophe, this man's brother came posting into the barrack-square with an order for his discharge, the very morning on which he was buried." (P. 522.)

"There are two modifications of corporal punishment employed in the navy, which naturally add much to its severity; in what is termed *flogging round the fleet*, the culprit is conveyed in a boat, with certain ceremonies, from ship to ship, receiving in succession a proportion of lashes from the boatswain's mate of each, while the respective crews are paraded to witness the infliction. In the punishment of the *gauntelope*, usually termed gantlet, the whole ship's crew is disposed in two rows, standing face to face on both sides of the deck, each man being furnished with a hard twisted cord, having several knots on it. The culprit, stripped to the waist, passes between these rows, each man being enjoined to apply the knottle to his shoulders as he moves along in slow time, preceded by the master-at-arms, who places a naked cutlas under his arm pointing backwards, by which means the delinquent is prevented from quickening his pace, and evading the punishment awarded him. These last-mentioned punishments are only inflicted for very heinous crimes; they are of a most severe and protracted nature; men occasionally fall down insensible under them, and they demand the most anxious attention from the medical officer whose distressing duty it may be to superintend them." (P. 525.)

The common method of dressing the backs of punished men in this country is with a solution of acetate of lead, applied by means of a cloth, or piece of lint, soaked in it; and, under this application, the swelling and ecchymosis consequent upon the injury for the most part subside rapidly, and the part speedily heals. It is difficult to determine the number of lashes a man may receive without endangering his life, but it is mentioned by Dr. B., as a remarkable fact, that, in cases where death has occurred, it has generally been from punishments of limited extent.

The volume concludes with an account of the diseases of troops on foreign stations.

